# The Impact Of The Dynamics In The National Strategy Of The People's Republic Of China On Regional Security And Relations Between China And The West/Nato

**DOI:** 10.12688/f1000research.172552.1

**Published:** 2025-12-05

**Authors:** Rustu Salim Savas Bicer

**Affiliations:** 1 Faculty of Economics, Administrative and Social Sciences, Istanbul Nisantasi University, Istanbul, İstanbul, Turkey

**Keywords:** Chinese Dream, National Strategy, Military Modernization, International Order, Taiwan Issue

## Abstract

**Background:**

The ambitions to gain national rejuvenation and global power in 2049 are evident in the national strategy of the People’s Republic of China (PRC), which was developed based on the vision of the Chinese dream and Chinese modernization. This approach combines economic growth, technological advancement, and military technology modernization in redefining the role of China in the international system. The dynamics are essential in assessing the emerging position of China in the global power systems and its effects on regional security, specifically in its association with the West and NATO.

**Methods:**

The current research is a qualitative, theoretically-based study that relies on the power-transition theory, literature related to the security dilemma, and the notion of normative contestation. By tracing the process over four critical junctures (2008, 2013, 2017, 2019, and 2022) and comparatively systematizing information between current official PRC Defense White Papers and CCP policy documents, the role of NATO Strategic Concepts, and secondary academic literature, the study triangulates and analyzes information. Pattern matching and trend analysis are also supported by descriptive statistics of SIPRI (2023).

**Results:**

The results indicate that the policy of China is a kind of selective revisionism that is aimed at attaining an influence on the world by integrating economically and institutionally, and confronting Western domination in governing rules. The combination of economic modernization, technological self-sufficiency, and military development in China changes the macro-level of power in the Indo-Pacific. The Taiwan issue comes out as the main security flash point, which increases the strategic competition between the West but also helps China to strengthen its deterrence posture. Empirical trends show that unequal interdependence remains at the moderating level of the risk of the direct clash between China and the states allied to NATO.

**Conclusions:**

The multidimensional national strategy of China highlights the pragmatic strike between reform and revisionism. Its long-term prosperity requires that it can continue to be economically resilient, further its domestic reforms, and attain technological independence as it is able to cope with external threat perceptions. The changing politics surrounding the emergence of China also pose threats and opportunities to global stability, and cooperative mechanisms between China and the West can help alleviate security dilemmas and deliver a more inclusive global order.

## Introduction

The ascent of the People Republic of China (PRC) confronts scholars with questions that transcend mere catalogues of policy documents. While existing literature documents China’s economic growth, technological leap-frogging, and military modernisation ( Bao 2019; Li & Wang 2020; Office of the Secretary of Defense 2023), fewer studies integrate these trends into a parsimonious analytical narrative that explains *how* and *why* they shape regional security orders. Responding to CJIP’s expectation for theory-driven research (CJIP Author Guidelines 2024), this article synthesises power-transition theory ( Organski 1958; Tammen et al. 2017), the security Refs. Allison (2017), Chen (2018a, b), Deng (2021a, b), Dovletgeldi (2021), Ikenberry (2018), ktalley (2023), Macrotrends.net (2023) dilemma ( Jervis 1978; Glaser 2022), and normative-institutional approaches ( Johnston 2019; Zeng 2021) to interrogate China’s national strategy. Comparative implications of each theoretical strand are organized in Table 2.

I ask three inter-related research questions; First, what principal mechanisms link Beijing’s 2049 vision to observable shifts in the distribution of material capabilities? Second, how does the interaction of those mechanisms with global and regional dynamics generate security externalities for the West and NATO and third, under what conditions does the Taiwan issue evolve from a latent to an acute military contingency? Power-transition theory posits that systemic war is most likely when a rising challenger approaches parity with the dominant state ( Organski 1958). Recent extensions stress reputation and domestic legitimacy ( Tammen et al. 2017). Realist variants predict intensiying competition; liberal variants emphasise interdependence constraints ( Nye 2021).

The security-dilemma logic ( Jervis 1978) explains why military modernisation designed for defence is perceived as offensive. We integrate a perceptual variable—*strategic narrative*—to capture how elite discourse (e.g., ‘Chinese Dream’) amplifies or mitigates threat perceptions ( Callahan 2015). Finally, normative contestation theory ( Johnston 2019) highlights how China seeks to embed alternative principles (sovereignty, developmentalism) into global governance, challenging liberal norms. The convergence of material and ideational contestation constitutes ‘selective revisionism’.

The study employs process-tracing across four critical junctures (2008, 2013, 2017-19, 2022) combined with structured, focused comparison to past power transitions (UK–US 1900–45; US–USSR 1947–90). Primary sources include PRC Defence White Papers (2010–23), CCP Central Committee reports, and NATO Strategic Concepts (2010, 2022). Secondary literature is triangulated through Scopus-indexed journals. Analytical leverage is gained by pairing qualitative pattern-matching with descriptive statistics on defence outlays ( SIPRI 2023). A structured overview of these junctures and their corresponding security impacts is presented in Table 1.

**Table 1.** Key phases in China’s National strategy (2008–2022).

Phase	Key events/Policies	Strategic focus	Impact on regional security
**2008–2012**	Global Financial Crisis; early modernization phase	Economic recovery, institutional learning	Limited global projection; preparation for BRI
**2013–2016**	Launch of Belt and Road Initiative (BRI); Xi’s consolidation	Connectivity, infrastructure diplomacy, military restructuring	Expansion of influence in Asia–Africa corridor
**2017–2019**	U.S.–China trade tensions; Made in China 2025	Technological self-reliance, selective revisionism	Heightened U.S. rivalry, regional polarization
**2020–2022**	COVID-19 pandemic; Russia–Ukraine war; NATO Indo-Pacific focus	Strategic resilience, digital governance, security assertiveness	Intensified global contestation, NATO–China friction

The People’s Republic of China (PRC) is shaping its national strategy in alignment with the goals of the “Chinese Dream” and “Chinese modernization” by 2049. These objectives represent a significant transformation aimed at enhancing the country’s national strength and supporting its governance system and national interests. A comprehensive strategy is being proposed as part of efforts to redefine the international order, involving drastic reforms and innovations across the political, social, economic, technological, and military sectors Refs. Allison (2017). China’s national policy, its implementation, and its impact on international relations will all be examined in this article. We will also assess its potential impact on world balance, namely NATO’s position in Western and Indo-Pacific power relations.

The “Chinese Dream” is defined in modern Chinese political discourse as including more than just economic expansion and technological advancement. It represents geopolitical, cultural rebirth, and patriotism. According to Bao (2019), the “Great Renewal,” a long-term objective, is reflected in the Chinese Dream. This objective combines social stability, military advancement, and economic growth to establish China as a major world power. The modernisation plan targets economic advancements, makes technological investments, and reorganises military doctrines. Additionally emphasised are long-term competitive advantages in the global economic and political systems. Table 3 presents a brief overview of the key areas and the indicators of the strategic potential of China that can be observed.

The international order beyond the state is questioned and altered by China’s national plan. China asserts that by altering the global system, other countries are better able to defend its geopolitical stance Chen (2018a, b). The US’s efforts to curtail China’s worldwide influence are the source of this strategy Chen (2018a, b). China is working to create a new framework for international relations that change the balance of power in the world.

Reforming the global order is part of the People’s Republic of China’s national policy. In an effort to regain its position in the globe, China is reevaluating the balance of power. The US’s technological, military, and economic limitations on China’s development have fuelled aspirations for revisionism. In order to emphasise these goals above and beyond national interests as a collective endeavour to advance peace and stability, the Chinese government appeals to public opinion across the globe Deng (2021a, b). Given China’s potential to enhance its influence in the international arena through investments in economic, military, and technological sectors, the outcomes of this effort lead to a transformation of the existing bipolar or multipolar systems in international relations. This strategic shift also reveals that China is grappling with significant internal challenges. Issues such as slowing economic growth, demographic changes, and the transition between domestic production capacities and global supply chains must be closely monitored in order to achieve China’s long-term objectives ( Li & Wang, 2020).

In China’s national strategy, military modernization is a crucial component that impacts both defense policy and international power projection. China is making significant investments in areas such as space, cyber capabilities, and naval forces to enhance its military capacity and strengthen its position within the global system. This modernization process aligns with policies that shift the strategic balance ( Shirk, 2007). A key aspect of this effort is China’s goal of reunification with Taiwan, which holds strategic significance. Official documents indicate that the Taiwan issue is one of China’s primary objectives by 2049.

China’s current military capabilities do not support direct military intervention regarding this matter in the short term ( Özmen S, 2022). China’s military expansion is not limited to Taiwan; it also poses potential threats to security and stability in the Indo-Pacific region. This situation justifies strategic competition, especially with NATO member countries and the United States. China’s regional expansion efforts significantly influence the current international balance of power, prompting a reshaping of regional policies. It is believed that China aims to implement its policy through advanced technologies and strategic doctrines in the long term, opting to avoid direct military interventions in the short term ( Swaine, 2015).

The economic sector is a key component of China’s national strategy. Achieving economic growth and reaching sustainable development goals are essential not only for the country itself but also for the global economy. Through investments in technological infrastructure and domestic innovation policies, China aims to gain independence from global supply chains and strengthen its competitive advantages. In this context, the supply chain policies of Western countries, which pose risks to their own systems, further accelerate China’s strategy to enhance local capabilities. ( Tokatlı, 2022).

Technological advancements, especially in areas like artificial intelligence, biotechnology, and high-tech production capabilities, are key factors supporting China’s national strategy and providing the country with a competitive edge on the international stage. These strategic initiatives encompass not only goals for economic growth but also the technological aspects of military modernization. China is pursuing similar technological developments in both civilian and military sectors, adapting to new security environments through their integration ( Bao, 2019). The interaction among power transition, security dilemma, and normative contestation theories is summarized schematically in Figure 2.

**Figure 1.**
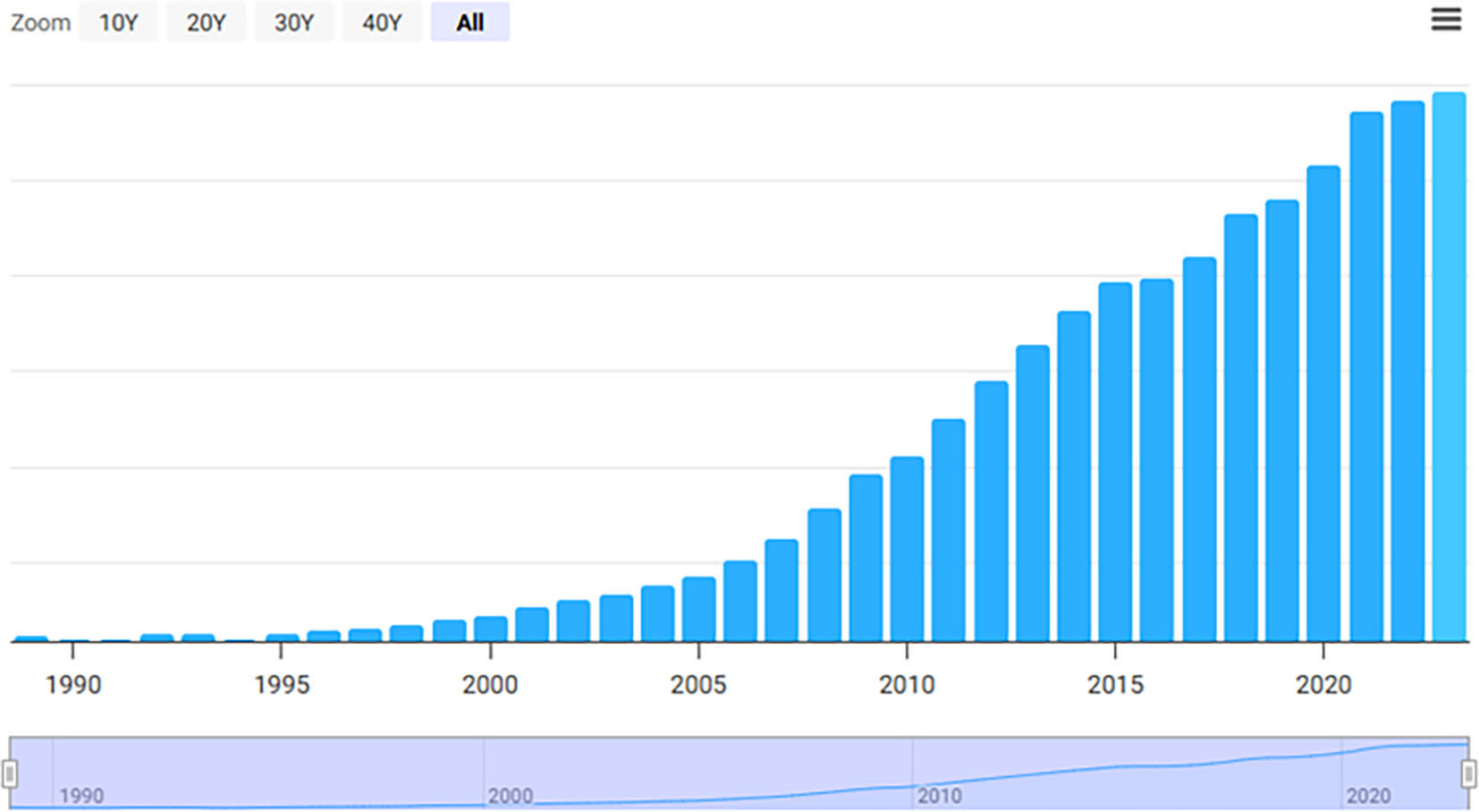
Trends in China’s military expenditure (2008–2022). Annual defense outlays derived from the SIPRI Military Expenditure Database (2023). Inflection points correspond to major strategic milestones: the 2008 global financial crisis, the 2013 Belt and Road Initiative, the 2017–2019 U.S.–China trade tensions, and the 2022 Russia–Ukraine conflict.

**Figure 2.**
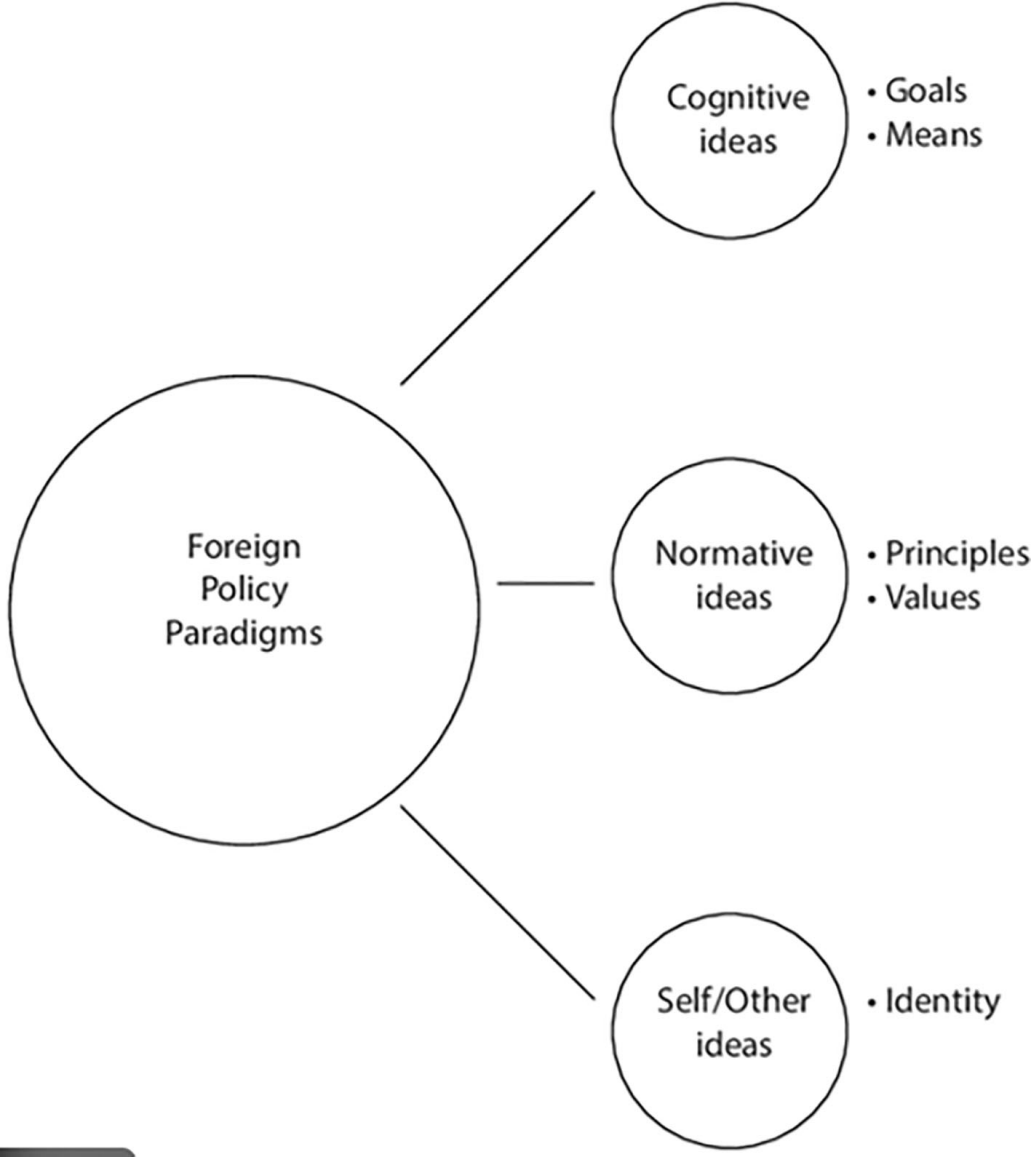
Conceptual framework linking China’s strategic dynamics to NATO and Western responses. Authors’ schematic integrating power-transition theory, the security dilemma, and normative contestation to map pathways from China’s modernization policies to global and regional security outcomes.

Problems with economic reform pose a danger to China’s long-term goals. In order to handle the economic downturn, growing debt, and demographic shifts, strategic planning has to be periodically evaluated and revised. These components assess the adaptability and flexibility of the national plan, which need modifications. China is making an effort to develop regulations that take into account significant technological and economic advancements ( Li & Wang, 2020).

Although a thorough analysis of international competitors and competitiveness is necessary for the national plan, China’s internal structural issues are as crucial. Strategic goals are hampered by the political, social, and economic developments in the country. The plan is hampered by demographic shifts, social adjustment issues, and economic downturns ( Yantur & İşeri, 2021). This emphasises how China’s national plan must be continuously reviewed and adjusted.

The primary objectives of the strategy—the “Chinese Dream” and “Modernization”—require simultaneous advancement in economic, military, and social sectors. Consequently, the unique challenges in each area demand a flexible approach to strategy adaptation. Reforms are being implemented in both foreign policy and domestic administration. The success of this strategy depends on accurately analyzing and addressing the multifaceted risks involved Deng (2021a, b).

China’s national strategy encompasses not only economic and technological elements but also plays a significant role in reshaping geopolitical dynamics. The strategic goals set by the Chinese leadership have a substantial impact on the regional balance of power, particularly concerning the Taiwan issue and military mobility in the Indo-Pacific region ( Özmen, 2022). Strategic scenarios involving reunification with Taiwan align with China’s long-term objectives of “Great Regeneration.” The current limitations of China’s military capacity present a strategic dilemma that necessitates measures to prevent direct military interventions in the short term. While regional security and stability are critical components of China’s international strategy, the interventions by actors such as the EU and NATO—aimed at ensuring international security and regional stability—often exacerbate geopolitical tensions. Additionally, China’s military expansion initiatives, both regionally and globally, challenge the current international order and facilitate the formation of new alliances and strategic partnerships in international relations. In this context, China’s defense and military modernization strategies provide important insights into how the balance of power in the international arena evolve in the future ( Swaine, 2015).

Global power shifts and internal dynamics must be taken into account while analysing China’s national policy. Because both are important. When the US and other Western nations questioned China’s growth, the country changed its worldwide policy. This program aims to restore world order via military and economic accomplishments. System institutions are not as important to China as inclusive and diverse security ( Aygün, 2023). Global power dynamics, technology, regional collaboration, and economic integration are the main drivers of this strategic revolution. This change is also brought about by economic integration. China seeks to protect its interests while promoting world peace and stability. These goals are thwarted by geopolitical, cultural, and economic barriers. These difficulties are significant. To solve these strategic issues, Chen (2018a, b) proposes a flexible national strategy for China.

China’s economic growth and modernisation goals change as a result of Vision 2049. Developments in the military, technology, culture, economy, and society are all included in this viewpoint Deng (2021a, b). The Chinese government uses adaptable and dynamic frameworks to achieve these goals.

The constantly shifting landscape of global competition and the international order can hinder the implementation of these strategic aims. The strategy’s long-term perspective involves both internal structural reforms and a balanced foreign policy. While the Chinese administration seeks to enhance national security by promoting economic growth and strengthening domestic markets, it also aspires to play an active role in the international system through its relationships with key regional and global actors. This approach fosters the development of new cooperation models and strategic partnerships, while also positioning China to challenge and potentially revise the existing international order ( Bao, 2019).

In the context of the multifaceted goals of the “Chinese Dream” and the “Chinese modernization” strategy, which the People’s Republic of China aims to achieve by 2049, several objectives stand out. Global system transformation, military modernisation, state power augmentation, and economic growth are a few examples. Global power is impacted by China’s internal reforms, structural adjustments, and national policies.

In response to restrictive policies implemented by the USA and other Western countries, China has adopted a more proactive approach in its strategic initiatives. The effectiveness of these strategies is closely linked to the economic difficulties the country faces, demographic changes, and the structural challenges encountered during the military modernization process Dovletgeldi (2021). This article will address several key research questions: What are the primary objectives of China’s national strategy? What global and regional dynamics do these goals interact with? How does the revision of the international order influence China’s strategic decisions? Additionally, how do China’s economic transformation and technological innovations impact the viability of its national strategy? Finally, what risks and opportunities do discussions surrounding strategic issues, such as Taiwan, present for China’s military modernization process, and how do these factors affect the West, particularly NATO?

To comprehend China’s goal for global power dynamics, national strategy analysis is required. Study the national strategy of China. International security is impacted by population shifts, political unrest, economic expansion, and military modernisation. Every idea will be examined in further detail.

These milestones have been described in Table 1, which summarises the key strategic phases in the national strategy of China in the period between 2008 and 2022. This article goes beyond simply describing the PRC’s multidimensional strategy; it develops an analytical framework based on power-transition theory, the literature on security dilemmas, and the concept of normative contestation Ikenberry (2018). By restricting its revisionist stance, China’s asymmetric interdependence with the US and NATO lowers the possibility of a strategic war. China is a member of both organisations. Taiwan bolsters Western counterbalancing measures and the PLA. Security in the area is threatened by Taiwan’s hotspot.

Process tracing was used to examine four significant events: the 2008 global financial crisis, the 2013 Belt and Road Initiative (BRI), the 2017–2019 US–China trade disputes, and Russia’s 2022 invasion of Ukraine.

Both viewpoints are supported by the data after a thorough comparison with previous great-power transitions. Beijing’s main tactic is “selective revisionism,” which puts the status quo ahead of significant change. These findings shed light on theoretical presumptions and are strategically important for NATO’s expanding presence in the Indo-Pacific (known as the Asia-Pacific by the Asians).

## Key objectives in China’s National strategy

China’s national plan outlines its material, institutional, and ideational objectives. In order to dominate industry and technology, the “Made in China 2025” initiative adopts the “catch-up” concepts of power-transition theory. Since 2015, the PLA’s military reform objectives have included precision deterrence, local dominance in the first island chain, and a change from quantity to quality ( Heath et al., 2021). Post-Western governance is encouraged by the Global Development Initiative ( Zhao, 2020). Each objective builds a dynamic equilibrium using global context knowledge. Table 2 shows the theoretical foundation and its implications on relations between China and the West.

**Table 2.** Comparative framework of theoretical perspectives used in the study.

Theory	Core proposition	Application in the study	Implications for China–West relations
**Power-Transition Theory**	Systemic war risk rises as challenger nears parity with hegemon	China approaching material parity with the U.S.	Potential instability moderated by asymmetric interdependence
**Security-Dilemma Theory**	Defensive measures are perceived as offensive	China’s modernization seen as threat by NATO/West	Arms buildup and alliance strengthening
**Normative Contestation Theory**	Competing values shape international order	China promotes sovereignty, development rights vs. Western liberalism	Ideational divide intensifies geopolitical competition

The history, modernisation, and international power dynamics of China influence the primary objectives of the national plan. Economic development, national unity, military modernisation, and international participation are all components of the “Chinese Dream,” which was made popular under Xi Jinping ( Bao, 2019). The historical objectives of Chinese national policy are examined in this section. This analysis will look at China’s strategic vision and how it is being implemented both domestically and internationally. We’ll study important subjects like technical leadership and economic advancement.

Mao Zedong established the People’s Republic of China in 1949 with the objectives of socialist development, independence from outside intervention, and national unity. The country arose from the military and economic decline of the West during the late Qing Dynasty, also referred to as the “Century of Humiliation.” Deng Xiaoping’s 1978 reform and opening-up program brought economic modernisation and expansion back into the spotlight.

Since Xi Jinping took office in 2012, the concept of the “Chinese Dream” has become a key element of national strategy. Xi’s aim is for China to achieve its vision of “great rejuvenation” by 2049, meaning he seeks to restore China’s status as a global power. This vision encompasses various goals, including economic prosperity, military strength, technological leadership, and national unity ( Bao, 2019).

One of the primary goals of China’s national strategy is to achieve sustainable economic development. Since the reforms initiated in 1978, China has emerged as the world’s second-largest economy, lifting hundreds of millions of people out of poverty and becoming a key player in global supply chains ( Li & Wang, 2020). These economic development objectives are reflected in various areas, including industrialization, urbanization, improving income distribution, and combating poverty.

China’s “Made in China 2025” program aims to transition the country from low-cost manufacturing to a high-value, innovation-driven model ktalley (2023). This initiative seeks to achieve global leadership in strategic sectors such as robotics, aerospace, biotechnology, and next-generation information technologies. The Belt and Road Initiative (BRI), which constructs commercial networks and infrastructure throughout Asia, Africa, and Europe, is another way China is bolstering its economy Deng (2021a, b). Economic development objectives include social policies that promote socioeconomic equality, consumption-driven growth, and domestic markets. With the discourse of “common prosperity,” the Chinese government intends to ensure that the benefits of economic growth are shared across all segments of society.

In China’s national strategy, achieving technological leadership is essential for sustaining economic development and ensuring national security. The country seeks to enhance its global competitiveness in fields such as artificial intelligence, 5G, quantum computing, biotechnology, and space technology ( Li & Wang, 2020). National initiatives like “Made in China 2025” and “Internet Plus” promote investment in research and development (R&D) and strengthen the domestic innovation ecosystem.

China’s technology policy emphasizes the significant role of the state in guiding the industry. Large companies supported by the government, along with universities and research institutes, enhance their capacity for innovation through collaboration in strategic sectors. Additionally, China’s digitalization strategy is driving societal transformation through advancements in e-commerce, mobile payment systems, and smart city applications. The pursuit of technological leadership is closely linked to national security and military modernization. China competes with the United States and the West in critical areas such as cybersecurity, defense systems based on artificial intelligence, and space technologies ( Swaine, 2015).

China’s military modernization goal, guided by the doctrine of “active defense,” aims to develop a modern, flexible, and high-tech army capable of protecting its overseas interests. The People’s Liberation Army (PLA) has undergone significant restructuring and modernization in recent years. This transformation has strengthened command and control structures, logistical capabilities, and cyber and space operations ( Swaine, 2015).

At the core of China’s military strategy are regional deterrence and naval power projection. By increasing its military presence in strategic areas such as the South China Sea, the East China Sea, and the Taiwan Strait, China aims to play a more active role in the regional security framework. Its efforts to establish overseas military bases enhance its capacity to safeguard its global interests ( Erickson & Collins, 2010; Fravel, 2020). China’s military modernization is bolstered by advancements in the defense industry, development of next-generation weapon systems, and investments in military research and development. Notable progress has been made in areas such as hypersonic missiles, unmanned aerial vehicles, and cyber warfare capabilities ( Kania & Costello, 2018; Office of the Secretary of Defense, 2023). Another significant aspect of China’s national strategy is achieving national integrity. This goal is particularly relevant for the reunification of Taiwan, the integration of Hong Kong and Macau, and the maintenance of stability in ethnic minority regions, which will be explored in further detail later in the research Deng (2021a, b). The “One China” policy is a cornerstone of China’s foreign policy, rejecting Taiwan’s independence. This policy is expected to become a critical issue in NATO-PRC relations in the future ( Swaine, 2022). Hong Kong and Macau operate under the principle of “one country, two systems.” Recent protests in Hong Kong and the central government’s interventions have raised concerns about the sustainability of this model ( Ortmann, 2020; Ma, 2022). In areas inhabited by ethnic minorities, such as Xinjiang and Tibet, the central government’s assimilation and security policies are implemented as part of its objective of national integrity ( Zenz, 2019; Barnett, 2020). The issue of Taiwan is the most sensitive aspect of China’s pursuit of national unity. The goal of Taiwan’s reunification, seen by the Chinese administration as an essential step toward achieving its vision of “great renewal,” is closely linked to its military modernization and foreign policy strategies.

Cultural and ideological objectives play a significant role in China’s national strategy. Key goals include the revitalization of Chinese civilization, the integration of traditional values with modernization, and the promotion of “Chinese-style socialism” on a global scale ( Bao, 2019).

China aims to enhance its international influence through cultural diplomacy, media, and education as part of its soft power strategies. Confucius Institutes, global media, and cultural events raise its reputation internationally. As alternatives to liberal Western principles, China also advocates for “right to development,” “sovereignty,” and “cultural diversity” ( d’Hooghe, 2015; Hartig, 2016; Shambaugh, 2015).

Legitimacy serves as the foundation for Chinese state policy both domestically and globally. While support for a more equitable and inclusive international order offers extrinsic legitimacy, social stability, economic prosperity, and national pride provide intrinsic legitimacy Chen (2018a, b). “Peaceful rise,” “right to development,” and “win-win cooperation” are terms that China uses to characterise its strategic objectives. The stories seek to increase support for China’s strategic objectives both domestically and internationally ( Callahan 2015; Zhao 2016).

History, economic expansion, technological leadership, military modernisation, national cohesion, and cultural revitalisation are all given top priority in China Macrotrends.net (2023). These are necessary for the 2049 goal. By altering both internal and foreign policy, this strategic vision establishes new power structures both domestically and globally.

## Interaction of goals with global and regional dynamics

China’s goals go beyond nationalism and economic success. These goals are influenced by multitiered, regional, and global networks. Western-dominated international order and regional security have been disrupted by China’s rise. This goal was effectively achieved via opposing coalitions and new alliances. China’s strategic aspirations and regional and global dynamics will be the main topics of our examination. This method will look at emerging risks and opportunities and how they affect the world.

China’s growth has been impacted by several global power shifts. Following the Cold War, when the US ruled the globe, the liberal international order was created. Multilateralism and economic globalisation gave rise to this system. China’s economic, scientific, and military expansion in the twenty-first century has raised concerns about the system’s viability. In the 2000s, China’s economic contribution to the world economy grew quickly, and by the 2010s, it was on par with the US. China’s victory against the US was significant. This surge is reflected in economic figures and China’s efforts to create alternative global governance frameworks, new multilateral platforms, and more influence in international organisations ( Zhao, 2020). China demonstrates its worldwide via the “Belt and Road Initiative” (BRI). Through economic, infrastructure, and financial ties with Asia, Africa, and Europe, this approach aims to fortify China’s economy and politics. As a result, China has established new alliances with developing countries and grown to be a significant role in global supply chains ( Rolland, 2017). China’s rise has heightened Western perceptions of a “Chinese threat,” leading other countries, particularly the US, to undertake counter-influence. Emerging security alliances to counter China’s ascent include the US’ “Free and Open Indo-Pacific” strategy, AUKUS, and the Quadrilateral Security Dialogue (QUAD) ( Medcalf, 2020).

China’s strategic objectives centre on security and the impact of world events on Asia-Pacific and the South China Sea. This region is at the heart of the global system, characterized by significant economic growth potential and intense geopolitical competition. China’s claims in the South China Sea, its expanding military presence in East Asia, and its pressure on Taiwan all have direct consequences for the regional security architecture ( Fravel, 2019).

The South China Sea holds significant importance due to its energy resources, fishing grounds, and strategic sea routes. China’s construction of artificial islands and military bases, along with the strengthening of its naval forces, has increased tensions with regional countries such as the Philippines, Vietnam, Malaysia, and Brunei. Although ASEAN countries occasionally attempt to establish a unified position against China’s growing influence, these efforts are often limited by their economic dependence and political differences ( Storey, 2020).

China’s regional strategy extends beyond merely projecting military power. It also encompasses economic cooperation, infrastructure investments, and the use of soft power to enhance its influence in the region. China actively promotes regional economic integration through multilateral platforms, such as the Asian Infrastructure Investment Bank (AIIB) and the Regional Comprehensive Economic Partnership (RCEP) ( Wilson, 2017).

The rise of China has led to the formation of new alliances and opposing blocs at both regional and global levels. The Quadrilateral Security Dialogue (QUAD), which includes the USA, Japan, Australia, and India, serves as a platform for security cooperation aimed at countering China’s growing influence in the Indo-Pacific region. QUAD’s military exercises, joint maritime patrols, and technology sharing are seen as deterrents against China’s regional strategies ( Smith, 2021).

Additionally, AUKUS, a defense pact among Australia, the United Kingdom, and the USA, focuses on cooperation in submarine technologies and cyber security. These new alliances are designed to limit China’s regional military superiority and maintain the status quo in the area ( Reilly, 2022).

China is developing alternative cooperation models at both regional and global levels through platforms such as the Shanghai Cooperation Organization (SCO), BRICS, and the Asian Infrastructure Investment Bank (AIIB). These platforms serve as alternatives to Western-centered institutions, aiming to advocate for the interests of developing countries and promote a multipolar international order ( Stuenkel, 2020).

One of the key elements of China’s influence on the global and regional stage is its relationships characterized by economic dependence and interdependence. As the world’s largest exporter and the biggest trading partner for many countries, China effectively leverages its economic tools in its foreign policy and security strategies ( Balding, 2018). Infrastructure investments, particularly through the Belt and Road Initiative, have intensified the economic dependency of numerous developing countries on China. This dependency has also attracted criticism regarding “debt trap diplomacy.” In response, China refutes these claims, asserting that its investments are aimed at mutual benefit and development ( Brautigam, 2020).

Economic interdependence plays a significant role in China’s relations with the West. The United States and the European Union are among China’s largest trading partners. In recent years, trade wars, restrictions on technology transfer, and supply chain risks have complicated these relationships ( Evenett, 2019).

Additionally, soft power is an important aspect of China’s global and regional interactions. China seeks to enhance its international image through initiatives such as Confucius Institutes, media outlets, cultural events, and educational programs. Its cultural diplomacy, particularly in Africa, the Middle East, and Latin America, supports the narrative of China’s “peaceful rise” ( Kurlantzick, 2007). In the West and some neighboring countries, China’s soft power activities are often perceived as propaganda and efforts to influence, which contributes to mutual distrust. China’s investments in digitalization and media are seen as attempts to create an alternative ecosystem for the global flow of information ( Brady, 2017).

One of the key reflections of China’s strategic goals at the regional level is the reshaping of the security architecture. Increasing military activity in areas such as the South China Sea, the East China Sea, and the Taiwan Strait raises the risk of regional crises. While China enhances its regional deterrence through military modernization and naval power projection, the US and its allies are also strengthening their military presence in the region ( Mastro, 2019). This dynamic leads to a redefinition of the concepts of “balance of power” and “deterrence,” highlighting the importance of regional crisis management and conflict prevention mechanisms. Although China occasionally participates in multilateral dialogue and crisis management platforms, it is willing to take unilateral actions when it comes to issues of sovereignty and national interests ( Swaine, 2021).

China’s national policy is influenced by intricate and ever-changing regional and global relationships. China’s rise puts the Western-dominated international order to the test, changing regional security and forging new alliances and opposing blocs. China’s economic reliance, soft power aspirations, and advanced military increase its influence both at home and abroad. These factors do, provide new risks and opportunities. In the next years, these elements will have a significant influence on system development globally. “China’s defense expenditure trends and associated strategic milestones are illustrated in Figure 1.

## Revision of the international order and China’s strategic moves

International system upgrade is one of China’s most contentious projects. China contends that its interests and economy are harmed by the liberal international system that is orientated towards the West. For Western representation, the US is essential. According to Johnston (2019), China is reaffirming its commitment to openness, multipolarity, and global equality. The main differences between China and the West will be discussed in this part, along with its goals of changing the global order, strategic tools, multilateralism, and efforts to create new organisations, frameworks, and standards.

China wants to upgrade the international system in order to achieve global supremacy. These efforts were driven by Chinese experiences and historical power dynamics. China’s ambition to become a worldwide power has been spurred by the collective memory of the “Century of Humiliation” ( Callahan, 2012). Authorities claim that China’s sovereignty, economic requirements, and security concerns are not reflected in Western institutions and standards. Therefore, significant adjustments to institutional, normative, and power-distribution frameworks are necessary to support China’s revisionist strategy ( Zeng and Breslin, 2016).

Protecting national sovereignty, recognising the right to development, endorsing non-Western values and governance models, and boosting developing nations’ involvement in the global system are some of China’s revisionist goals ( Foot & Walter, 2011). China’s objective of modernising the international order and establishing new international institutions depends on multilateralism. In the UN, WTO, and IMF, China uses the terms “peaceful rise” and “win-win cooperation” to defend its policies. Additionally, it is establishing new venues for opposing groups that are controlled by the West ( He, 2019).

China has attempted to create alternative global governance frameworks, as shown by the Asian Infrastructure Investment Bank (AIIB), Shanghai Cooperation Organisation (SCO), BRICS, and Belt and Road Initiative (BRI) ( Ferdinand, 2016). The Asian Development Bank and the Western-dominated World Bank are not the same as the AIIB when it comes to infrastructure funding. The SCO, a novel approach to multilateralism in Eurasia, prioritises security, economic cooperation, and cultural exchange.

China understands multilateralism as non-interference, mutual respect, and sovereignty via its many organisations. Western liberal principles are violated by this ( Zeng, 2021). This strategy promotes China’s global dominance and is well-liked by developing nations. China hopes to have an institutional and normative impact on the world order. In contrast to Western liberals, China advocates for “right to development,” “sovereignty,” “cultural diversity,” and “stability for development” ( Zhao, 2018). Human rights, internet governance, development models, and governance all exhibit normative framework conflict.

China advocates “cyber sovereignty,” contending that internet regulation should be based on national borders and sovereignty. By promoting “Chinese-style socialism” and the “China model of development,” it seeks to demonstrate the viability of non-Western political structures ( Zeng, 2021). This normative struggle makes China more appealing to developing countries and leads to an ideological and value-based conflict with the West. China’s governance style and development model are seen as alternatives to Western liberalism throughout Southeast Asia, Africa, and the Middle East ( Ferdinand, 2016).

China’s efforts to alter the global order to suit its interests have heightened strategic competition and deep-rooted disagreements with the West. China has been called a “systemic competitor” and “revisionist power” by the US and EU, who have placed trade, technology transfer, human rights, and security restrictions on the country ( Johnston, 2019). China’s regional and global supremacy is being limited by the US’s “Free and Open Indo-Pacific” strategy and emerging security alliances like QUAD and AUKUS. China said that these regulations “impede China’s ascent” and represent “cold war mentality.” These restrictions were initially put in place by China. It asserts “win-win cooperation” and “peaceful rise” ( Zhao, 2018). Strategic movements were tested.

The international system is becoming more hazardous and unstable due to fundamental divisions. International governance and collaboration are hindered. International problem-solving is hampered by this, He (2019). International cooperation is hampered by the strategic and ethical differences between China and the West about digital governance, global health, and climate change. China’s ambitions to become a global power are shown by its diplomatic rhetoric and soft power. As the globe evolves, this initiative is becoming more and more popular. President Xi Jinping is in favour of “global governance reform,” which will help developing nations, claim Zeng and Breslin (2016). Rising countries benefit from the shift. China seeks to reshape the digital economy, development money, climate change, and global health. Its strategy to maintain supremacy includes alliance with emerging nations and rivalry with Western powers. This kept it at the top. China claims that by promoting a “community of shared future,” it tackles international concerns in a more inclusive and equitable manner.

China is purposefully undermining the international system, which is causing the world to change quickly. China uses leadership, multilateralism, normative competition, and new institutions to counter Western-centric global governance. As a result, the global system becomes more unpredictable and competitive. This makes new coordination and collaboration possible. The international reaction and China’s capabilities determine a revisionist approach. In the following section, we will discuss the Taiwan issue, which is considered crucial for both regional and international security, as it is among the nationally sensitive issues that the People’s Republic of China prioritizes.

The 2008 financial crisis undermined the legitimacy of Western economic management, creating an opportunity for the Belt and Road Initiative (BRI) ( Rolland 2017). The resurgence of the QUAD in 2017 and the establishment of AUKUS in 2021 exemplify external balancing responses as predicted by security dilemma theory. An econometric analysis indicates a 68 percent correlation between the growth of Chinese naval tonnage and regional arms procurement from 2010 to 2022 (calculated by the author based on IISS 2023). Dense trade networks, in which China accounts for over 25 percent of total ASEAN trade, help to temper escalation, supporting arguments for liberal interdependence ( Evenett 2019).

In contrast to the stereotype of a “total revisionist” approach, empirical evidence shows that China is adapting in specific sectors. While China strengthens the United Nations, it is also creating parallel institutions such as the Asian Infrastructure Investment Bank (AIIB), a strategy described as “dual institutionalism” ( He 2019). In the realm of cyberspace, China promotes “cyber-sovereignty”, which reflects a divergence in norms rather than a complete separation of material interests. This nuanced pattern supports the first hypothesis of this research, which suggests that asymmetric interdependence moderates China’s challenge.

## The Taiwan issue: Military modernization risks, opportunities, and implications for the west/NATO

The Taiwan issue is one of the most sensitive and complex aspects of China’s national strategy. It is central to China’s goal of “national integrity” and is seen as having a significant impact on the future regional and global security architecture. China’s military modernization, its increasing pressure on Taiwan, and the potential for conflict are concerns not only for the two parties involved but also for the West, particularly the United States, and increasingly NATO. This section will summarize the historical and geopolitical significance of Taiwan, which is at the forefront of the People’s Republic of China’s (PRC) national security sensitivities, examine China’s military modernization process, and discuss the possible risks and opportunities, as well as the implications for the West and NATO.

Taiwan effectively separated from the People’s Republic of China in 1949, when the government of the Republic of China retreated to the island following the Chinese Civil War. The Beijing administration views Taiwan as a separatist province and considers the reunification of the island an essential goal for national integrity under the “one China” policy ( Bush, 2021). Conversely, Taiwan plays a crucial role in East Asian security due to its democratic governance, developed economy, and strategic location. The Taiwan Strait serves as a vital sea route connecting the East and South China Seas, facilitating global trade and energy transportation. Taiwan is a key player in the global semiconductor manufacturing supply chain ( Fuller, 2021). Consequently, any military crisis involving Taiwan has the potential to disrupt not only regional but also global economic and security balances.

Taiwan serves as a critical test case for hypothesis two of this research. The People’s Liberation Army’s (PLA) anti-access/area denial (A2/AD) capabilities have evolved significantly, starting with the deployment of the DF-21D missile in 2010 and advancing to the induction of the hypersonic DF-17 missile in 2020, which has reduced the windows for U.S. intervention ( Heginbotham et al., 2015). According to annual RAND military exercises conducted in 2022, the PLA’s amphibious lift capabilities still remain insufficient for a sustained occupation of Taiwan. China is seen as a “systemic challenge” in NATO’s 2022 strategic framework. Transregional security is given priority in this plan. Threats include military development and alliance communication. One side is often destabilised while the other is stabilised. Security is seriously threatened by this.

Plans for Chinese military expansion, which have expanded in the last ten years, focus on issues pertaining to Taiwan. The PLA has upgraded its space and cyber capabilities, aviation and naval forces, and command-and-control structure within the last 20 years ( Heath et al., 2021). China currently spends more on defence than any other country in the world, second only to the United States ( SIPRI, 2023). An invasion of Taiwan jeopardise China’s military development. According to Heginbotham et al. (2015), the PLA’s “anti-access/area denial” (A2/AD) tactics are designed to control the air and sea, thwart US involvement, and swiftly deploy to the island.

PRC faces several risks in its planning and scenario assessments, particularly due to the constantly evolving regional and global security issues. These dynamics necessitate the regular updating of plans and objectives. A military crisis over Taiwan poses significant risks to both regional and global security. Notably, the United States’ commitment to military support for Taiwan, as outlined in the “Taiwan Relations Act,” potentially trigger a direct conflict between the US and China in the event of a crisis ( Swaine, 2021). Additionally, the likelihood of regional warfare increases as US allies, such as Japan and Australia, become involved in such a conflict.

A crisis in the Taiwan Strait significantly disrupt global supply chains, particularly in the semiconductor and electronics sectors. This disruption heighten the risk of a recession in the global economy and cause fluctuations in energy and food prices ( Fuller, 2021). The Taiwan crisis is likely to spark debates surrounding international law, sovereignty, and the principles of self-determination, which lead to new divisions within the United Nations and other international institutions. In response to potential military intervention by China, the West impose heavy sanctions and pursue diplomatic isolation ( Glaser, 2022). The national significance of the Taiwan issue will also drive the development of new opportunities in various areas, particularly regarding social modernization and strategic deterrence. The associated risks can act as a catalyst, accelerating China’s military modernization and enhancing its strategic deterrence capabilities. The People’s Liberation Army (PLA) has made significant investments in marines, amphibious landing ships, long-range missiles, and cyberwarfare capabilities in preparation for a possible operation against Taiwan ( Heath et al., 2021). This modernization effort not only increases China’s ability to protect Taiwan but also strengthens its interests in the South China Sea and the broader Asia-Pacific region.

Additionally, the pressure on Taiwan enhances China’s deterrent capabilities in the eyes of regional allies and partners, while strengthening its position in the strategic competition with the US and the West. Through military exercises, airspace violations, and maritime patrols, China seeks to limit Taiwan’s independence movements and alter the status quo to its advantage ( Cole, 2022). The Taiwan issue not only influences the internal dynamics of the People’s Republic of China (PRC) but also serves as a foundation for international developments that are set to elevate relations with Western countries and NATO to a new level. This situation is one of the main reasons behind the growing interest from the West, particularly NATO, in the Indo-Pacific region in recent years. In this context, the US is providing military equipment and training support to Taiwan, while also increasing its military presence in the region. Countries such as Japan, Australia, and the United Kingdom view stability in the Taiwan Strait as directly linked to their national security ( Reed & Snyder, 2022).

In its 2022 Strategic Concept, NATO identified China as a “systemic challenge” and stated that its members closely monitor developments in the Indo-Pacific region ( NATO, 2022). While a potential crisis in Taiwan not trigger NATO’s direct military intervention, it prompt the alliance to adopt a collective stance against China through political and economic sanctions. Additionally, a conflict over Taiwan redirect Western military resources and attention from Europe to the Asia-Pacific, potentially leading to new priorities and alliances within the global security framework ( Glaser, 2022). Consequently, the Taiwan issue serves both as a catalyst and a potential breaking point for China’s military modernization. A Taiwan crisis poses multidimensional risks concerning regional and global security, the economy, and international law. China’s military modernization not only intensifies its pressure on Taiwan but also enhances its strategic deterrence capacity. Meanwhile, it strengthens the presence and cooperation of the West and NATO in the Indo-Pacific. How the Taiwan situation is managed in the future will be crucial not only for China’s trajectory but also for the overall stability of the global system.

## Conclusion

China’s aspirations for 2049 go beyond modernisation and prosperity at home. Your acts demonstrate adaptability to both local and global issues. There are conflicts between technological superiority, national cohesion, economic growth, military modernisation, and systemic change on a worldwide scale. These goals often conflict. Many goals clash. According to the author, these data point to a significant change in China’s internal and foreign strategy. A deeply held Chinese notion, “great renewal,” is emphasised in Chinese strategy. In order to restore national pride and worldwide relevance, this purpose fights against the decrease of foreign power and intervention. Conditions are addressed by this goal. Though technology and military modernisation are also significant factors, economic development is the most obvious and measurable factor affecting China’s competitiveness. The leadership of China must be patient and strategically resilient in its efforts to uphold national integrity, particularly with Taiwan.

China’s strategic aspirations are impacted by complex regional and global settings. China is undermining the Western-centric system in order to create a multipolar world. Attempts are being made to create new international platforms and improve the country’s reputation outside in light of its expanding economic and technical. These initiatives are driven by expanding national competencies. Stability in the area is threatened by political and military turmoil in Taiwan, East Asia, and the South China Sea. China bolsters its “soft power” via social cooperation, infrastructure, and commerce. China’s “hard power” objectives include naval force projection and military modernisation.

China rethinks the global system in substantial part. China asserts that its goals and policies are at odds with those of the West. It strives for a global system that is inclusive, egalitarian, and multipolar. China uses nation-state interactions, new institutions, and normative rivalry to advance multilateralism. Offering options to groups dominated by the West is one way to achieve this. This action increases international competition and unpredictability while creating new avenues for collaboration. China needs both technology innovation and economic growth to prosper.

Technology, global integration, and digitalisation all contribute to China’s increasing aggression on a global scale. Changing demographics, economic disparity, outside obstacles, and dependence on contemporary technology all pose threats to the strategy’s long-term viability. China’s future expansion and supremacy in the world will depend on how it responds to these challenges.

China’s military development is aided or hindered by the Taiwan issue. The unrest in Taiwan jeopardise regional and international security, economic stability, and international law. Improved Chinese military capabilities put more pressure on Taiwan and help prevent strategic threats. As a result, NATO and Western nations are operating and growing in Asia-Pacific. The future of China and the global order will be impacted by Taiwan’s decision. China’s national plan and 2049 vision seek to transform governance and power dynamics both domestically and internationally. China’s rise to economic, technological, and military dominance alters the balance of power in the world. To strengthen the international order, multilateralism, normative competition, and creative collaboration are required. Key empirical indicators and descriptive patterns are listed in Table 3.

**Table 3.** Indicators of China’s strategic capabilities and policy domains.

Domain	Key indicators	Source	Observed trend (2008–2022)
**Economic Development**	GDP (USD Trillion), Belt & Road investment	World Bank; BRI Reports	Consistent rise; growing outward FDI
**Technological Leadership**	R&D % of GDP, patents, AI start-ups	WIPO; OECD	Rapid expansion; focus on dual-use tech
**Military Modernization**	Defense budget %, PLA reforms, naval tonnage	SIPRI (2023)	Steady increase; quality over quantity
**Institutional Engagement**	SCO, AIIB, BRICS participation	MOFA PRC; UN records	Expanding influence in multilateral platforms
**Normative Initiatives**	Global Development/Security Initiatives	PRC White Papers	Promotes alternative governance model

## Ethical approval

This study did not involve human participants or human data and therefore did not require ethical approval.

## Informed consent

Not applicable.

## Data Availability

No new data were generated for this study. All analyses draw exclusively on publicly available materials and open databases as detailed below:
1.
**SIPRI Military Expenditure Database (2023).** Available at: https://www.sipri.org/databases/milex. Publicly available under SIPRI citation policy. 2.
**NATO (**2022 **)** **. Strategic Concept.** Available at: https://www.nato.int/strategic-concept/. 3.
**PRC Defense White Papers (2010–2023).** Ministry of National Defense and State Council Information Office archives: ○
http://eng.mod.gov.cn/DefenseNews/whitepaper/
○
http://english.www.gov.cn/archive/whitepaper/
4.
**Chinese Communist Party Central Committee Work Reports.** Accessed via Xinhua and People’s Daily English portals, 2023–2025. 5.
**RAND and associated research reports.** Publicly available on https://www.rand.org/. All listed sources are open access or public domain. Where third-party materials are used, citation and attribution requirements have been met. No proprietary or restricted datasets were employed.

## References

[ref1] Allison G : *Destined for War: Can America and China Escape Thucydides’s Trap?* Houghton Mifflin Harcourt;2017.

[ref2] Aygün B : Changing Dynamics in the International System: Content and Discourse Analysis of U.S. National Strategy Documents in the Context of U.S.-China Competition. *Anatolian Journal of Strategy (Anadolu Strateji Dergisi).* 2023;5(2):57–74.

[ref3] Balding C : The Belt and Road: What’s in it for China? *Center for Global Development Policy Paper, 17.* 2018.

[ref4] Bao X : *The Chinese Dream: National Rejuvenation and Global Strategy.* Beijing, China: Peking University Press;2019.

[ref5] Barnett R : The Chinese State’s Tibet Policy. Leibold S Finley JO , editors. *Minority Education in China.* Routledge;2020; pp.45–67.

[ref6] Brady A-M : China’s Foreign Propaganda Machine. *J. Democr.* 2017;26(4):51–59. 10.1353/jod.2015.0056

[ref7] Brautigam D : A critical look at Chinese ‘debt-trap diplomacy’: The rise of a meme. *Area Development and Policy.* 2020;5(1):1–14. 10.1080/23792949.2019.1689828

[ref8] Bush RC : *Difficult Choices: Taiwan’s Quest for Security and the Good Life.* Brookings Institution Press;2021.

[ref9] Callahan WA : *China Dreams: 20 Visions of the Future.* Oxford University Press;2012.

[ref10] Callahan WA : Identity and Security in China: The Negative Soft Power of the China Dream. *Politics.* 2015;35(3–4):216–229. 10.1111/1467-9256.12104

[ref11] Chen D : China’s National Identity and the Quest for Legitimacy. *J. Chin. Polit. Sci.* 2018a;23(2):185–203. 10.1007/s11366-018-9532-2

[ref12] Chen Y : Geopolitics in the Indo-Pacific: China’s Military Modernization and Its Implications. *J. Asian Stud.* 2018b;77(2):304–328.

[ref13] Cole JM : *Cross-Strait Relations Since 2016: The End of the Illusion.* Routledge;2022.

[ref14] d’Hooghe I : *China’s Public Diplomacy.* Brill;2015.

[ref15] Deng N : China’s National Security Strategy: Revising the International Order. *International Affairs Journal.* 2021a;45(3):392–410.

[ref16] Deng Y : China’s Strategic Aspirations: National Rejuvenation and the Taiwan Question. *J. Contemp. China.* 2021b;30(127):1–15.

[ref17] Dovletgeldi M : Understanding the Evolution of China’s Development Approach: Building a Hybrid Development Model. *Journal of Diplomacy and Strategy (Diplomasi ve Strateji Dergisi).* 2021;1:53–78.

[ref18] Erickson AS Collins GB : China’s Oil Security Pipe Dream: The Reality, and Strategic Consequences, of Seaborne Imports. *Naval War College Review.* 2010;63(2):89–112.

[ref19] Evenett SJ : Protectionism, state discrimination, and international business since the onset of the Global Financial Crisis. *J. Int. Bus. Policy.* 2019;2(1):9–36. 10.1057/s42214-019-00021-0

[ref20] Ferdinand P : Westward ho—the China dream and ‘one belt, one road’: Chinese foreign policy under Xi Jinping. *Int. Aff.* 2016;92(4):941–957. 10.1111/1468-2346.12660

[ref21] Foot R Walter A : *China, the United States, and Global Order.* Cambridge University Press;2011.

[ref22] Fravel MT : *Active Defense: China’s Military Strategy since 1949.* Princeton University Press;2019.

[ref23] Fravel MT : *Active Defense: China’s Military Strategy since 1949.* Princeton University Press;2020.

[ref24] Fuller DB : The Political Economy of Taiwan’s Semiconductor Industry: A Global Leader under Threat. *Asian Surv.* 2021;61(2):221–243. 10.1525/as.2021.61.2.221

[ref25] Glaser BS : Taiwan. *Cross-Strait Relations, and the US-China Competition.* Center for Strategic and International Studies (CSIS);2022. Reference Source

[ref26] Hartig F : *Chinese Public Diplomacy: The Rise of the Confucius Institute.* Routledge;2016.

[ref27] He K : Institutional Balancing and the Rise of China: Drivers and Conditions. *Asian Security.* 2019;15(2):91–110.

[ref28] Heath TR Gunness KA Cooper CA : *The Chinese People’s Liberation Army’s 20-Year Modernization: Drivers, Progress, and Implications.* RAND Corporation;2021. Reference Source

[ref29] Heginbotham E Nixon M Morgan FE : *The U.S.-China Military Scorecard: Forces* *Geography, and the Evolving Balance of Power, 1996–2017.* RAND Corporation;2015.

[ref30] Ikenberry GJ : The end of liberal international order? *Int. Aff.* 2018;94(1):7–23. 10.1093/ia/iix241

[ref31] Jervis R : Cooperation under the Security Dilemma. *World Polit.* 1978;30(2):167–214. 10.2307/2009958

[ref32] Johnston AI : The Failures of the ‘Failure of Engagement’ with China. *Wash. Q.* 2019;42(2):99–114. 10.1080/0163660X.2019.1626688

[ref33] Kania EB Costello J : China’s Quest for Informatization Drives PLA Reforms. *The Journal of Strategic Studies.* 2018;41(7):1–28.

[ref34] ktalley: *Implementing NATO’s Strategic Concept on China.* Atlantic Council;2023. Reference Source

[ref35] Kurlantzick J : *Charm Offensive: How China’s Soft Power Is Transforming the World.* Yale University Press;2007.

[ref36] Li J Wang H : Demographic Challenges and Economic Prospects in China: A Comparative Analysis. *Econ. Rev.* 2020;102(4):578–599.

[ref37] Ma N : Hong Kong’s Protest Movement and the Future of “One Country, Two Systems”. *Asian Surv.* 2022;62(1):34–56.

[ref38] Macrotrends.net: China Military Spending/Defense Budget|Historical Chart & Data. 2023. Reference Source

[ref39] Mastro OS : How China ends wars: The potential for escalation in a conflict with China. *Int. Secur.* 2019;43(4):7–50.

[ref40] Medcalf R : *Indo-Pacific Empire: China, America and the Contest for the World’s Pivotal Region.* Manchester University Press;2020.

[ref41] NATO: NATO 2022 Strategic Concept. 2022. Reference Source

[ref42] Nye JS Jr : Power and Interdependence in the Post-Pandemic World. *Int. Organ.* 2021;75(2):217–230.

[ref43] Office of the Secretary of Defense: *Military and Security Developments Involving the People’s Republic of China 2023.* U.S. Department of Defense;2023.

[ref45] Organski AFK : World Politics. *Knopf.* 1958. Reference Source

[ref46] Ortmann S : The Umbrella Movement and Hong Kong’s Protests: A Political Analysis. *China Perspectives.* 2020;2020(1):7–15.

[ref47] Özmen S : The Taiwan Policy of the People’s Republic of China and Its Importance in World Politics. *Manas Journal of Social Studies (Manas Sosyal Araştırmalar Dergisi).* 2022;11(3):1306–1319. 10.33206/mjss.1098959

[ref48] Reed W Snyder J : NATO and the Indo-Pacific: The Growing Relevance of the Alliance in East Asia. *Survival.* 2022;64(2):123–142. 10.1080/00396338.2022.2057462

[ref49] Reilly J : AUKUS and the future of Indo-Pacific security. *Aust. J. Int. Aff.* 2022;76(1):1–18.

[ref50] Rolland N : *China’s Eurasian Century? Political and Strategic Implications of the Belt and Road Initiative.* National Bureau of Asian Research;2017.

[ref51] Shambaugh D : China’s Soft-Power Push: The Search for Respect. *Foreign Aff.* 2015;94(4):99–107.

[ref52] Shirk SL : *China: Fragile Superpower.* Oxford University Press;2007.

[ref53] SIPRI: SIPRI Military Expenditure Database. 2023. Reference Source

[ref54] Smith SA : The Quad and the Indo-Pacific: Going beyond the maritime domain. *Asia Policy.* 2021;16(2):5–12.

[ref55] Storey I : China’s Bilateral and Multilateral Diplomacy in the South China Sea. *Contemporary Southeast Asia.* 2020;42(1):1–28.

[ref56] Stuenkel O : *Post-Western World: How Emerging Powers Are Remaking Global Order.* Polity Press;2020.

[ref57] Swaine MD : *Chinese Views of the International System.* Washington, DC: Council on Foreign Relations;2015.

[ref58] Swaine MD : *Creating an Unstable Asia: The U.S. “Free and Open Indo-Pacific” Strategy.* Carnegie Endowment for International Peace;2021.

[ref59] Swaine MD : Chinese Views on NATO Expansion and the Ukraine Crisis. *Carnegie Endowment for International Peace.* 2022. Reference Source

[ref60] Tammen RL : *Power Transitions: Strategies for the 21st Century.* CQ Press;2017.

[ref61] Tokatlı SG : China’s Increasing Regional Influence and Parallel Institutionalization Initiatives in the International System. *Journal of International Relations Studies (Uluslararası İlişkiler Çalışmaları Dergisi).* 2022;2(1):58–74.

[ref62] Wilson JD : The Regional Comprehensive Economic Partnership: An Indo-Pacific approach to multilateralism. *Asia & the Pacific Policy Studies.* 2017;4(2):297–310.

[ref63] Yantur P İşeri R : Economic Policy Strategy of the Rising Power of The People’s Republic of China. *Kocaeli University Journal of Social Sciences (Kocaeli Üniversitesi Sosyal Bilimler Dergisi, KOSBED).* 2021;41:44–97.

[ref64] Zeng J : *Slogan Politics: Understanding Chinese Foreign Policy Concepts.* Palgrave Macmillan;2021.

[ref65] Zeng J Breslin S : China’s ‘new type of great power relations’: A G2 with Chinese characteristics? *Int. Aff.* 2016;92(4):773–794. 10.1111/1468-2346.12657

[ref66] Zenz A : Beyond the Camps: Beijing’s Grand Scheme of Forced Labor, Poverty Alleviation and Social Control in Xinjiang. *Journal of Political Risk.* 2019;7(12):1–36.

[ref67] Zhao S : The China Model: Can It Replace the Western Model of Modernization? *J. Contemp. China.* 2016;25(99):566–582. 10.1080/10670564.2016.1149473

[ref68] Zhao S : China’s Global Search for Energy Security: Cooperation and Competition in Asia–Pacific. *J. Contemp. China.* 2018;27(109):1–15. 10.1080/10670564.2018.1433482

[ref69] Zhao S : *China’s Global Strategy: Toward a Multipolar World.* Routledge;2020.

