# Supplementary material for: The Impact Of The Dynamics In The National Strategy Of The People's Republic Of China On Regional Security And Relations Between China And The West/Nato
Source: F1000Res. 2025 Dec 5;14:1366. [Version 1] doi: 10.12688/f1000research.172552.1 (PMC13146458; doi:10.12688/f1000research.172552.1)
Supplement: Peer review history

# Reviewer report: Reviewer response for version 1

**DOI:** 10.5256/f1000research.190288.r448046

**Published:** 2026-05-05

**Authors:** Bama andika putra

**Affiliations:** 1 Universitas Hasanuddin Fakultas Ilmu Sosial dan Ilmu Politik, Makassar, South Sulawesi, Indonesia

The article does a commendable job in explaining the unique dynamics associated with China's national strategies, and how it relates/affects regional security. Through the qualitative methods, the utilization of the power-transition theory allows for a more nuanced understanding on the role of NATO within the context of China and its regional security.

 The narratives introduced are convincing, outlining the importance of conceptualized strategies in shaping regional order, which can easily run counter to the existing status quo. Nevertheless, I would expect several changes to enhance the author's discussion. 

 First, on the point of regional security, there is little emphasis on the regional security situation with regard to China. The mentioning of the South China Sea, for example, lacks in-depth analysis of empirical data and relevant case studies assessed by past scholars. I'd recommend the following readings to enhance the discussions on contemporary developments of the South China Sea:

 (refer 1,2,3)

Is the work clearly and accurately presented and does it cite the current literature?

Yes

If applicable, is the statistical analysis and its interpretation appropriate?

Yes

Are all the source data underlying the results available to ensure full reproducibility?

Yes

Is the study design appropriate and is the work technically sound?

Yes

Are the conclusions drawn adequately supported by the results?

Yes

Are sufficient details of methods and analysis provided to allow replication by others?

Yes

Reviewer Expertise:

International Relations

I confirm that I have read this submission and believe that I have an appropriate level of expertise to confirm that it is of an acceptable scientific standard.

## References

[1] : Lawships or warships? Coast guards as agents of (in)stability in the Pacific and South and East China Sea. *Marine Policy* .2022;140: 10.1016/j.marpol.2022.105048 10.1016/j.marpol.2022.105048

[2] : Campaigns And Drones: Maritime Security In The South China Sea. *Security science journal* .2025;6(1) : 10.37458/ssj.6.1.1 7-18 10.37458/ssj.6.1.1

[3] : Making waves: marine research vessels in China and Vietnam’s overlapping maritime zones. *Frontiers in Political Science* .2025;7: 10.3389/fpos.2025.1670797 10.3389/fpos.2025.1670797

# Reviewer report: Reviewer response for version 1

**DOI:** 10.5256/f1000research.190288.r462345

**Published:** 2026-03-05

**Authors:** Sertif Demir

**Affiliations:** 1 University of Turkish Aeronautical Association, Ankara, Turkey

This comprehensive article provides a sophisticated analysis of the People’s Republic of China’s (PRC) strategic evolution toward its 2049 "Great Rejuvenation" goals. By skillfully synthesizing power-transition theory, security dilemma logic, and normative contestation, the author moves beyond mere policy description to offer a parsimonious analytical narrative. The core concept of "selective revisionism" serves as a vital bridge between China’s economic integration into the global system and its simultaneous challenge to Western-centric rules and norms. This theoretical framework is robustly supported by a qualitative process-tracing methodology that examines critical junctures from 2008 to 2022, effectively triangulating primary CCP policy documents with NATO strategic concepts and SIPRI military expenditure data.

 The research offers a significant contribution to the field by demonstrating how China’s military modernization and technological self-sufficiency are not isolated trends, but interconnected mechanisms that redefine the Indo-Pacific security architecture. A notable strength of the study is the high degree of consistency between its research questions and its empirical results; for instance, the analysis of the Taiwan issue clearly illustrates the transition from latent to acute strategic competition. Furthermore, the article provides a balanced conclusion: while China's rise amplifies the security dilemma, the moderating effect of "asymmetric interdependence" offers a nuanced view on the potential for stability.

 Overall, the work is a logically structured, methodologically sound, and theoretically grounded piece that is well-suited for high-level academic publication, offering valuable insights into the shifting macro-level distribution of global power.

Is the work clearly and accurately presented and does it cite the current literature?

Yes

If applicable, is the statistical analysis and its interpretation appropriate?

Not applicable

Are all the source data underlying the results available to ensure full reproducibility?

No source data required

Is the study design appropriate and is the work technically sound?

Yes

Are the conclusions drawn adequately supported by the results?

Yes

Are sufficient details of methods and analysis provided to allow replication by others?

Yes

Reviewer Expertise:

International relations, Turkish Foreign Polcy, International Security, İNternational Politics

I confirm that I have read this submission and believe that I have an appropriate level of expertise to confirm that it is of an acceptable scientific standard.

# Reviewer report: Reviewer response for version 1

**DOI:** 10.5256/f1000research.190288.r439894

**Published:** 2026-01-20

**Authors:** Nguyễn Anh Cường

**Affiliations:** 1 University of Social Sciences and Humanities, Hanoi, Vietnam

General assessment

 The manuscript addresses an important and timely topic: how the evolution of the PRC's national strategy is associated with regional security dynamics and China-West/NATO relations. The paper brings together power-transition theory, the security dilemma, and normative contestation, and it makes an effort to connect official PRC and NATO documents with an observable indicator (defence spending trends). The topic is suitable for a qualitative research article; however, as written, several elements required for scientific soundness and reproducibility are insufficiently specified.

 Major issues that should be addressed (required)

 1) Clarify and operationalise the research design. The Methods section states that the study uses process tracing across critical junctures (2008, 2013, 2017-2019, 2022) and a structured comparison, but the concrete analytic steps are not described. Please specify: (a) the exact corpus of primary documents (PRC Defence White Papers/CCP reports/NATO Strategic Concepts and other NATO documents) and inclusion criteria; (b) how evidence was extracted (coding scheme, keywords, sections of documents, inter-coder reliability if applicable); (c) what constitutes a 'critical juncture' in the design and how the causal mechanisms are tested; and (d) how the structured comparison to past power transitions is executed (case selection, variables compared, and what constitutes supporting or disconfirming evidence). An appendix listing the documents analysed and a short description of the coding/analysis protocol would materially improve replicability.

 2) Strengthen the link between claims and evidence. Several substantive claims (e.g., the strength of relationships between expenditure trends and strategic shifts, or assertions about causal effects on NATO posture and regional security) read as conclusions rather than findings. Please ensure each major claim is grounded in explicit evidence from the documents or clearly labelled as interpretation. Where causal language is used, discuss alternative explanations (e.g., domestic economic cycles, US policy shifts, crisis dynamics) and explain why the preferred mechanism is more plausible.

 3) Statistical/descriptive analysis needs transparency. If the manuscript uses descriptive statistics or correlation statements, the variables, data sources, time coverage, and calculation method must be reported (including any transformations). Otherwise, consider limiting the quantitative component to clearly-described descriptive trends (with source data provided).

 4) Improve the literature base and referencing. The manuscript cites some non-scholarly web sources and the reference formatting is inconsistent. Please replace non-academic sources with peer-reviewed literature or official documents, ensure all claims about prior work are properly cited, and update the literature review to reflect current scholarship on China-NATO relations and NATO's Indo-Pacific engagement.

 5) Provide source data for figures and tables. For Figure 1 and for author-constructed tables, please provide the underlying data used to produce the figure and the table inputs (e.g., SIPRI values by year; coded document excerpts or a structured evidence table), either as supplementary files or via a public repository, as per the journal's reproducibility expectations.

 Minor issues and presentation

 - Tighten the structure and reduce repetition: clearly state the contribution and research questions early, and ensure the Results section is organised to answer those questions.

 - Define key terms consistently (e.g., 'strategic narrative', 'selective revisionism', 'normative contestation') and ensure consistent time-period labels across sections and tables.

 - Language polishing is needed throughout (grammar, word choice, and clarity). A professional copy-edit would improve readability.

 - Expand the limitations section: discuss document-selection bias, lack of primary interviews, and the constraints of using expenditure as a proxy for strategic intent.

 Recommendation

 I recommend 'Approved with Reservations' provided that the authors substantially clarify the methods/analysis, improve evidentiary support for key claims, and supply the source data underlying figures and tables.

Is the work clearly and accurately presented and does it cite the current literature?

Partly

If applicable, is the statistical analysis and its interpretation appropriate?

Partly

Are all the source data underlying the results available to ensure full reproducibility?

Partly

Is the study design appropriate and is the work technically sound?

Partly

Are the conclusions drawn adequately supported by the results?

Partly

Are sufficient details of methods and analysis provided to allow replication by others?

No

Reviewer Expertise:

International Relations; Security Studies; China foreign and security policy; NATO strategy; Regional order in the Indo-Pacific/Euro-Atlantic interface.

I confirm that I have read this submission and believe that I have an appropriate level of expertise to confirm that it is of an acceptable scientific standard, however I have significant reservations, as outlined above.

# Response

**Published:** 2026-01-20

**Authors:** Rustu Salim Savas Bicer

**Affiliations:** International Relations, Istanbul Nisantasi University, Istanbul, Turkey

Thank you very much for your careful reading of the manuscript and for the detailed and constructive evaluation. I am grateful for your positive assessment of the relevance of the topic and the theoretical framework, and I particularly appreciate your thorough methodological and reproducibility-focused comments.

 I acknowledge the main concerns you raised regarding the need to clarify and operationalise the research design, including the specification of the document corpus, coding and evidence-extraction procedures, the definition of critical junctures, and the structured comparison framework. In the revised version, I will substantially expand the Methods section, add an appendix listing all primary documents analysed, and provide a clear description of the analytical protocol to ensure replicability.

 I also take note of your comments on strengthening the link between claims and evidence, improving transparency in the descriptive/statistical component, updating and standardizing the literature base, and providing the underlying data for figures and tables as supplementary material. These points will be addressed systematically in the revision.

 In addition, I will work on improving the structure, tightening the presentation, defining key concepts more consistently, expanding the limitations section, and undertaking professional language editing to enhance clarity and precision.

 Thank you again for your valuable guidance. Your suggestions will significantly improve the scientific rigor and transparency of the study, and I look forward to submitting a thoroughly revised version after the remaining reviewer reports are received.
